# The Efficacy and Safety of Adalimumab in Treating Pediatric Noninfectious Chronic Anterior Uveitis With Peripheral Retinal Vascular Leakage: A Pilot Study

**DOI:** 10.3389/fmed.2022.813696

**Published:** 2022-03-29

**Authors:** Hang Song, Chan Zhao, Junyan Xiao, Fei Gao, Donghui Li, Meifen Zhang

**Affiliations:** ^1^Department of Ophthalmology, Peking Union Medical College Hospital, Beijing, China; ^2^Key Laboratory of Ocular Fundus Diseases, Chinese Academy of Medical Sciences & Peking Union Medical College, Beijing, China

**Keywords:** adalimumab, pediatric uveitis, peripheral retinal vascular leakage, ultra widefield fluorescence fundus angiography, TNF antagonist

## Abstract

**Purpose:**

This study is aimed to assess the efficacy of adalimumab in alleviating peripheral vascular leakage in pediatric chronic anterior uveitis patients, along with its ability to improve best-corrected visual acuity (BCVA) and inflammation parameters, its efficacy in reducing topical glucocorticosteroids (GCs) and systemic immunomodulatory therapy (IMT), and its safety profile.

**Methods:**

A self-controlled study of pediatric chronic anterior uveitis patients who presented with peripheral retinal vascular leakage on ultra-widefield fluorescein fundus angiography and underwent adalimumab treatment was conducted. The primary outcome was the extent of retinal vascular leakage at the 3- and 6-month follow-up visits. Secondary outcomes included BCVA, inflammation parameters (fresh keratic precipitates, anterior chamber cell, and vitreous cell grades), frequency of topical glucocorticosteroid eye drops, IMT load, and adverse effects at the 3- and 6-month follow-up visits.

**Results:**

Twenty patients with a mean age of 9.30 ± 3.26 years old were included. The mean follow-up period was 9.0 ± 3.0 months, with all patients followed up for at least 6 months. At the 3- and 6-month follow-ups, the peripheral vascular leakage score decreased significantly (2.87, 95% CI (2.14, 3.60)*, p* < 0.001 for 3 months, 2.75, 95% CI (1.76, 3.73)*, p* < 0.001 for 6 months). Alongside BCVA (*p* = 0.013 for 3 months, *p* = 0.005 for 6 months) was improved significantly, inflammatory parameters represented by fresh keratic precipitates, anterior chamber cell, and vitreous cell grades were improved significantly (*p* < 0.001, *p* < 0.001, for all parameters) and topical GC usage was significantly reduced (*p* < 0.001, *p* < 0.001) at 3 and 6 months. There was also a statistically significant reduction in systemic IMT load at 6 months (*p* < 0.001). Adverse events in the observation period included local redness around the injection site and mild upper respiratory symptoms.

**Conclusion:**

Adalimumab could effectively alleviate peripheral vascular leakage in pediatric patients with chronic anterior uveitis. It could also be helpful in improving BCVA and inflammation parameters and decreasing topical glucocorticosteroid eye drops and systemic IMT. Adalimumab is generally safe for pediatric uveitis.

## Introduction

Pediatric uveitis, which usually presents as long-lasting, recurrent intraocular inflammation and may lead to early-onset visual disability, is one of the most challenging ocular conditions. Topical glucocorticosteroids (GCs) in combination with systemic immunomodulatory therapy (IMT), such as oral GC, methotrexate (MTX), or cyclosporin A (CsA), as needed have traditionally been the mainstay treatments for pediatric uveitis. However, long-term topical GC treatment increases the risk of developing complications, such as cataracts and glaucoma. The growth-inhibiting and weight-gaining effects of systemic GC and the variable efficacies and potential toxicities of IMT agents pose considerable concerns and call for better treatments for pediatric uveitis.

Biologics that target key inflammatory mediators represent a novel therapeutic modality for inflammatory diseases. Adalimumab, a humanized recombinant antibody directed against soluble and cell-bound tumor necrosis factor-alpha (TNF-α), has increasingly been recognized as a promising agent for treating noninfectious uveitis in adults and children (1–4). One of the most convincing studies regarding pediatric uveitis is the SYCAMORE study, a randomized, double blinded, placebo-controlled study that included 90 pediatric juvenile idiopathic arthritis (JIA)-associated uveitis patients, providing compelling evidence that adalimumab in combination with MTX was associated with a lower rate of treatment failure than MTX alone (3). However, further investigations are needed to shed more light on the indications, timings, dosages, and duration of adalimumab treatment and disease monitoring solutions in various pediatric uveitic conditions (5).

In our clinical observation, we found that children with chronic anterior uveitis often had peripheral vascular leakage detected by the ultra-widefield fluorescein angiography (UWFFA), even when there were no obvious vitreous cells or retinal lesions. In this study, we aimed to evaluate the efficacy of adalimumab in alleviating peripheral vascular leakage in pediatric chronic anterior uveitis, along with its ability to improve best-corrected visual acuity (BCVA) and inflammation parameters, its efficacy in reducing topical GC and systemic IMT, and its safety profile.

## Materials and Methods

### Oversight

A self-controlled study of pediatric patients (aged between 4 and 16 years old) with noninfectious chronic anterior uveitis who underwent adalimumab treatment in our center between May 2020 and July 2021 was conducted. Written informed consent was obtained from the parent of each participating child after being fully informed of the nature of the study, which was in accordance with the Declaration of Helsinki and approved by the Institutional Review Board of Peking Union Medical College Hospital (ZS-2788).

### Patients and Treatment Regimen

In regular outpatient clinics, children with noninfectious chronic anterior uveitis who had a body weight of ≥15 kg, a disease course longer than 3 months, and active inflammation after slit lamp examination were suggested to undergo UWFFA. Active inflammation was defined as more than 5 anterior chamber cells in 1-mm^2^ slit lamp beam field according to the Standardization of Uveitis Nomenclature (SUN) criteria (6). UWFFA was performed using Optos California (Optos PLC, USA). Two milliliters of sodium fluorescein were given intravenously, and angiograms centered on the posterior pole and superior, inferior, temporal, and nasal sweeps were captured. Frames were captured at close intervals in 0–3 min, followed by intermediate phase frames at approximately 7 min and late frames at approximately 15 min. If UWFFA for the intermediate phase frames suggested peripheral retinal vascular leakage (involving an area outside the retinovascular arcades but not the posterior pole), patients were considered for enrollment. Further screening tests for potential contraindications of adalimumab and baseline evaluation would be performed, such as blood cell count, liver functions, renal functions, hepatitis B virus, hepatitis C virus, and blood T-SPOT. tuberculosis (TB), antinuclear antibodies, and chest X-rays. Children with active TB, hepatitis B (HBV) and hepatitis C (HCV) infections, immune deficits, opportunistic infections, history of demyelinating disease of the central nervous system, and other severe chronic diseases were excluded. Children with eye surgery history within 3 months and complications that interfere with fundus observation were also excluded. There was usually a time interval between the time of determining inflammation exacerbation and waiting for the lab/UWFFA results back. Children were given traditional topical GC eye drops and systemic IMT treatment to control active inflammation during that time interval. After a full evaluation of the indication and contraindication, adalimumab was administered subcutaneously at 40 mg every 2 weeks if body weight ≥30 kg or 20 mg every 2 weeks if 15 kg ≤ body weight <30 kg. Topical GCs were gradually tapered at each follow-up visit depending on disease severity and the patients' responsiveness to treatment. If patients could tolerate topical GC dose reduction, systemic IMT would also be tapered. As patients' anterior chamber remained quiet for longer than 3 months and retinal vascular leakage healed, the interval of adalimumab administration was prolonged as appropriate based on the clinician's experience.

### Follow-Up and End Points

Follow-up visits were scheduled every 2 weeks in the active phase and every 1–2 months in the quiescent phase. A complete ophthalmic examination, such as BCVA, intraocular pressure (IOP), slit-lamp examination of the anterior segment and vitreous, and fundoscopy, was performed at each visit. UWFFA was conducted at the 3- and 6-month follow-up visits. If retinal vascular leakage healed without relapse of anterior chamber inflammation, UWFFA at 6 months was not performed. Complete blood count and renal and hepatic function were monitored every month for safety profiles.

**Figure 1:**
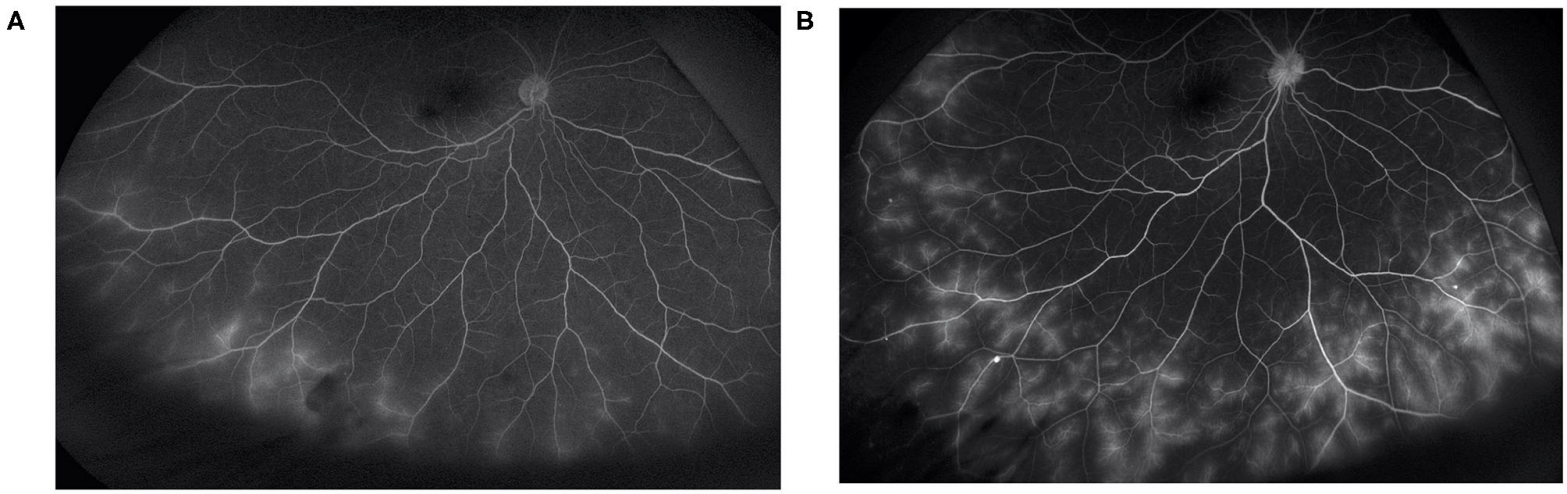
Demonstration of limited **(A)** and diffuse **(B)** vascular leakage.

Basic information collected included patient demographics, uveitis etiologies, course of the disease, prior history of topical GC and systemic IMT treatments, and ocular complications due to prolonged inflammation. The primary outcome was the extent of vascular leakage on UWFFA. The secondary outcomes were BCVA, inflammatory parameters, such as fresh keratic precipitates, anterior chamber cells and vitreous cells, topical GC eye drop frequency, and IMT (i.e., oral GC and immunosuppressive drug) load. The dosing and interval of adalimumab and the adverse events of adalimumab were also recorded. Peripheral retinal vascular leakage is quantified based on the method developed by the Angiography Scoring for Uveitis Working Group (ASUWOG) (7), in which vascular leakage in each peripheral quadrant is scored 1 if limited and scored 2 if diffuse, as demonstrated by Figure 1. Vascular leakage was evaluated by two ophthalmologists (H. S. and JY. X.) and the average score was calculated for analysis. Relapse was defined as a two-step increase in anterior chamber cell grade or an increase from 3 to 4 according to SUN criteria (6). Visual acuity was transformed to the logarithm of the minimum angle of resolution (logMAR) for data analysis. Fresh keratic precipitate was recorded in a dichotomous method. The anterior chamber cells were recorded by the SUN criteria, which count the anterior chamber cells per 1-mm^2^ field on standard slit-lamp examination (6). The slit lamp was pushed forward to the vitreous for vitreous cell evaluation. The grading method was the same as the anterior chamber cell grading described by SUN. Systemic IMT load is assessed with a weighted semiquantitative scale for each medication, as presented in Table 1, to provide a combined, single numeric score for the systemic immunosuppression load according to a method by Nussenblatt et al. (8).

**Table 1:** Grading scheme used to quantify immunomodulatory therapy (IMT) load.

**Medication**	**Immunosuppression grade (based on dose in mg/kg/day or per week if dosed weekly)**									
	**0**	**1**	**2**	**3**	**4**	**5**	**6**	**7**	**8**	**9**
Prednisone	0	<0.15	0.15–0.30	0.31–0.45	0.46–0.60	0.61–0.75	0.76–0.90	0.91–1.05	1.06–1.20	>1.2
Cyclosporine	0	<0.75	0.75–1.50	1.51–2.25	2.26–3.00	3.01–3.75	3.76–4.50	4.51–5.25	5.26–6.00	>6.00
Mycophenolate	0	<10	10–20	21–30	31–40	41–50	51–60	>60	-	-
Methotrexate	0	<0.05	0.05–0.10	0.11–0.15	0.16–0.20	0.21–0.25	0.26–0.30	31–0.35	0.36–0.40	>0.40

*Dose ranges are for the average in mg/kg/day, except for weekly doses of methotrexate, which are measured as mg/kg/week*.

### Statistical Analysis

Statistical analysis was performed using Statistical Package for the Social Sciences (SPSS, Chicago, IL, USA) version 23.0. A quantile-quantile plot was used for the test of normality. The ASUWOG score is presented as the mean ± SD and was analyzed with paired Student's t-test. Non-normally distributed continuous variables, such as LogMAR BCVA, systemic IMT load, and topical GC eye drop frequency, were described as medians (IQR) and compared with the Wilcoxon signed-rank test, in which the difference in the median value was compared with the Hodges–Lehmann method, calculated by the R statistics. Inflammatory parameters, such as fresh keratic precipitates, different grades of anterior chamber cell, and vitreous cell, were demonstrated as proportions of patients and analyzed with the chi-square test. For the primary outcome, the ASUWOG score, we compared the measurements at 3 and 6 months with the baseline measurement. We used Bonferroni's correction for multiple comparisons in the primary outcome, in which a two-sided *p* < 0.05/2 = 0.025 was regarded as statistically significant. For the secondary outcomes, the same correction was made within each outcome, but we did not further adjust the multiple comparisons among different secondary outcomes. Hence, findings from secondary outcomes were regarded as exploratory.

## Results

A total of twenty pediatric patients with chronic anterior uveitis were included, of which seventeen were idiopathic, two were associated with JIA, and one with Blau syndrome. At the beginning of the study, all patients were on topical GC or systemic therapy, or combined. Eighteen patients were using topical GC, six patients were using oral prednisone, and fourteen patients were on immunosuppressive therapy (Table 2). One patient was using etanercept in addition to both oral prednisone and MTX treatment. For complications before the application of adalimumab, eight patients had lens opacities, two patients had band keratopathy, five patients had posterior or anterior synechia, and one patient had to use topical eye drops for high IOP. The mean time from baseline UWFFA and the start of adalimumab was 8.87 ± 7.28 days, ranging from 0 to 28 days. The mean follow-up period after administration of adalimumab treatment was 9.0 ±3.0 months, with all patients followed up for ≥6 months. Table 2 summarizes the baseline characteristics of the included patients.

**Table 2:** Baseline features of the included patients when adalimumab was initiated.

**Parameters**	***N* (%)**
Age (mean ± standard deviation) (years)	9.30 ± 3.26
Sex (male/female)	15 (75)/5 (25)
Affected eyes (unilateral/bilateral)	1 (5)/19 (95)
Complications	12 (60)
Lens opacities	8 (40)
Band keratopathy	2 (10)
Posterior or anterior synechia	5 (25)
High intraocular pressure	1 (5)
Body weight (≥ 30 kg/ <30 kg)	15 (75)/5 (25)
Systemic IMT therapy	14 (70)
Prednisone + Methotrexate + Mycophenolate	1 (5)
Prednisone + Methotrexate + Cyclosporine	1 (5)
Prednisone + Methotrexate	4 (5)
Methotrexate + Cyclosporine	1 (5)
Methotrexate	6 (30)
Mycophenolate	1 (5)

**Figure 2:**
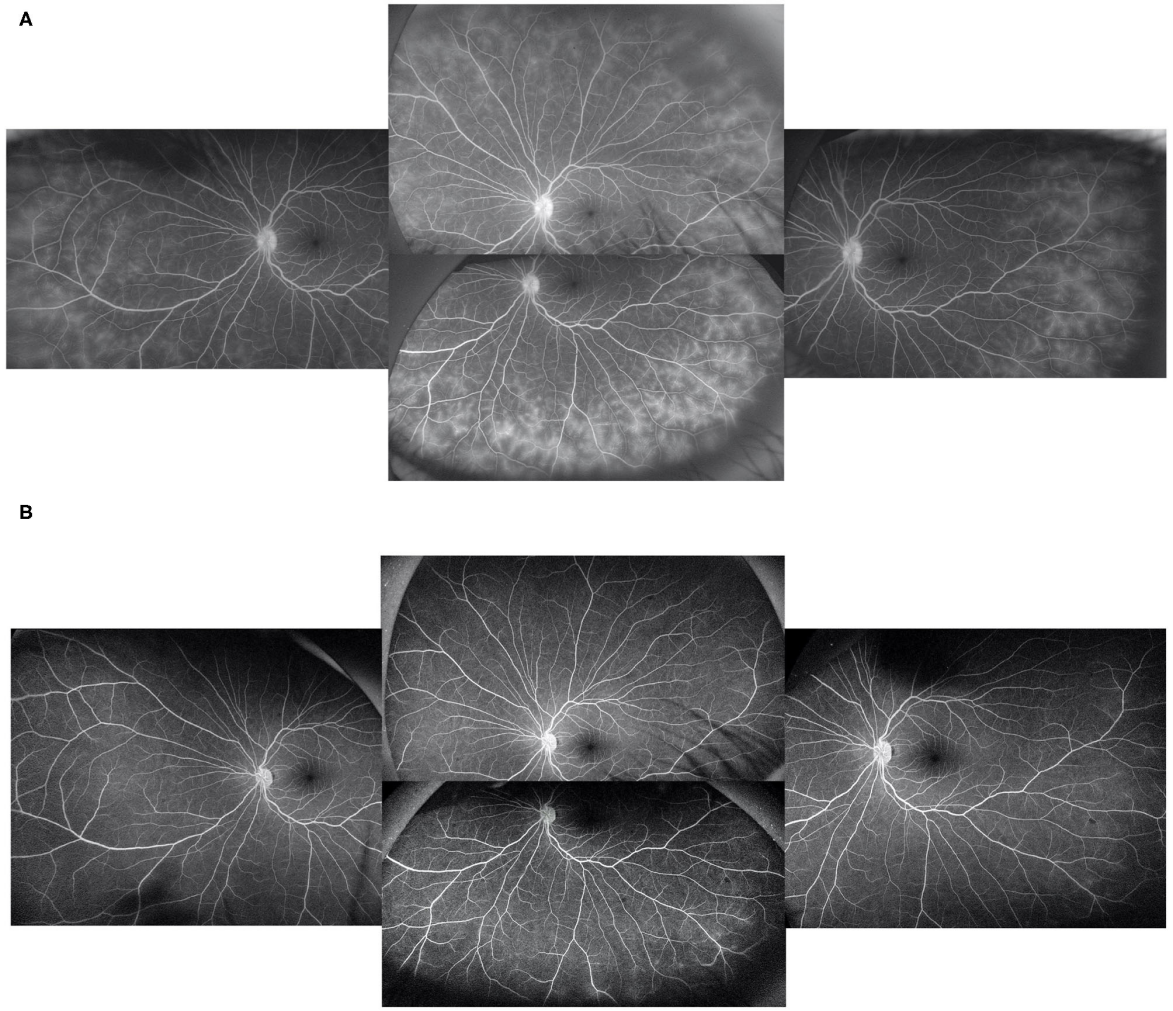
In a patient with peripheral vascular leakage at baseline **(A)**, 3 months of adalimumab treatment led to complete alleviation of vascular leakage **(B)**.

At baseline, the ASUWOG score was 4 ± 3. Compared to baseline, the ASUWOG score decreased significantly at the 3-month follow-up visit to 2 ± 2 (2.87, 95% CI (2.14, 3.60), *p* < 0.001; Figure 2). For patients who had a complete remission of vascular leakage and had no inflammatory exacerbation on slit lamp examination during regular follow-ups, UWFFA was not performed. Thus, only ten children underwent the UWFFA at the 6-month follow-up visit, and the ASUWOG score demonstrated a decrease in these patients from 5 ± 3 at baseline to 3 ± 2 at 3 months and further decreased to 2 ± 2 at 6 months (2.75, 95% CI (1.76, 3.73), *p* < 0.001, 6 months vs. baseline). At the time of adalimumab application, fifteen eyes achieved inflammatory remission with six to eight times 1% prednisolone eye drops per day. The remaining 24 eyes achieved inflammatory control after 3 months of treatment. Ocular inflammation parameters, such as fresh keratic precipitates, anterior chamber cell, and vitreous cell, were improved significantly at the 3-month follow-up visit (*p* < 0.001, *p* < 0.001, *p* < 0.001; Table 3). There was a statistically significant difference in improvements in BCVA (*p* = 0.013, *p* = 0.005) and a reduction in topical GC eye drops (*p* < 0.001, *p* < 0.001) at the 3- and 6-month follow-up visits. The systemic immunosuppression load was also reduced gradually but only showed a significant difference compared with baseline at 6 months (*p* = 0.404 for 3 months, *p* = 0.014 for 6 months; Table 4). All these parameters further improved at the additional last follow-up visit if available (Tables 3, 4).

**Table 3:** Main inflammatory outcomes under slit-lamp examination at baseline, 3, 6 months, and the last visit if longer than 6 months follow-up available after adalimumab initiation.

**Parameters**		**Baseline (*n =* 39)**	**3 months (*n =* 39)**	**6 months (*n =* 39)**	**Last visit (*n =* 26)**	**Odds ratio, 95% confidence interval and** ***P value***		
						**3 months vs. baseline**	**6 months vs. baseline**	**last visit vs. baseline**
Fresh keratic precipitates [No. (%)]		14 (35.90)	1 (2.56)	0 (0)	0 (0)	21.28, (2.63, 172.15) <0.001	23.08, (2.87, 185.38) <0.001	15.58, (1.92, 126.53) <0.001
Anterior chamber	0	15 (38.46)	35 (89.74)	34 (87.18)	24 (92.31)	Reference	Reference	Reference
cell [No. (%)]	0.5+	13 (33.33)	2 (5.19)	2 (5.19)	2 (7.69)	0.066, (0.007 to 0.360)	0.068, (0.014, 0.399)	0.096, (0.019, 0.487)
	1+	5 (12.82)	0 (0)	0 (0)	0 (0)	0.074, (0.008, 0.607)	0.076, (0.008, 0.686)	0.107, (0.012, 0.970)
	2+	6 (15.38)	0 (0)	0 (0)	0 (0)	0.067, (0.007, 0.560) <0.001	0.065, (0.007, 0.576) <0.001	0.091, (0.010, 0.815) <0.001
Vitreous cell [No. (%)]	0	8 (20.51)	23 (62.16)	32 (82.05)	23 (88.46)	Reference	Reference	Reference
	0.5+	13 (33.33)	15 (58.98)	3 (7.69)	2 (7.69)	0.401, (0.134, 1.200)	0.020, (0.002, 0.179)	0.043, (0.009, 0.200),
	1+	17 (43.59)	1 (2.56)	1 (2.56)	0 (0)	0.306, (0.106, 0.888)	0.015, (0.002, 0.128)	0.021, (0.002, 0.180),
	2+	1 (2.56)	0 (0)	0 (0)	0 (0)	0.188, (0.015, 2.330) <0.001	0.136, (0.011, 1.680) <0.001	0.188, (0.015, 2.330), <0.001

**Table 4:** Other secondary outcomes at baseline, 3, 6 months, and the last visit if longer than 6 months follow-up available after adalimumab initiation.

**Parameters**	**Baseline**	**3 months**	**6 months**	**last visit**	**Median difference, 95% confidence interval**		
**[median (IQR^†^)]**	**(*n =* 39)**	**(*n =* 39)**	**(*n =* 39)**	**(*n =* 26)**	**and** ***P value***		
					**3 months vs. baseline**	**6 months vs. baseline**	**Last visit vs. baseline**
LogMAR BCVA	0.00 (0.00, 0.00)	0.00 (0.00, 0.97)	0.00 (-0.79, 0.97)	0.00 (0.00, 0.00)	0.09, (-0.09, 0.15), 0.013	0.11, (0.04, 0.20), 0.005	0.10, (-0.4, 0.20), 0.013
IMT load	4.5 (0.0,7.0)	3.5 (0.0,6.0)	3.5 (0.0,6.0)	1.5 (0.0, 5.0)	1.0, (-1.5, 4.0), 0.439	3.0, (1.0, 4.5), 0.014	3.0, (2.0, 5.0), 0.058
Topical GC frequency^*^	4 (1,6)	0.5 (0, 0.5)	0.5 (0, 0.5)	0 (0, 0)	3.75, (2.75, 4.75), <0.001	4.0, (3.25, 5.00), <0.001	3.5, (2.25, 4.50), <0.001

† *IQR, interquartile range*;

* *1 represents 1% prednisolone acetate one drop per day, 0.5 represents 1% prednisolone acetate one drop every other day, or 0.5% loteprednol etabonate one drop per day*.

*BCVA, best corrected visual acuity; ASUWOG, Angiography Scoring for Uveitis Working Group; GCs, glucocorticosteroids*.

Adalimumab was prolonged to every 3 weeks in 15 patients, and the average time point of this prolongation was after 10.6 shots of adalimumab. Three out of the 15 patients prolonged the treatment interval to every month after an average of 14.3 shots. One patient stopped adalimumab after 20 shots without relapse. One patient developed a mild flare of uveitis at the 12-month follow-up visit 2 months after stopping 16 shots of adalimumab, presenting as an increase in anterior chamber cells from 0 to 1. At that time, the patient was under neither topical GC nor systemic IMT and was then added to topical GC eye drops. All other patients remained inflammatory quiescent in the available observation period.

Adverse events during the follow-up period included local redness around the injection site (1/20, 5%), a slight increase in uric acid and alanine aminotransferase (1/20, 5%), and common cold and bronchitis (5/20, 20%). Adalimumab was postponed for 3–5 days when the last condition was encountered. No patient had serious adverse events that led to the withdrawal of adalimumab.

## Discussion

Anterior uveitis is the most common form of pediatric uveitis and is prone to become chronic. Complications caused by recurrent or chronic inflammation and treatment, such as secondary glaucoma, complicated cataracts, and band keratopathy, often lead to permanent damage to ocular structures if not treated appropriately and in a timely manner (9, 10). Children display a relatively good response to topical GC but are more exposed to risks of drug-induced glaucoma (11). Growth retardation and weight gain caused by systemic GC are magnified in children. The first choice of immunosuppressor, MTX, could only reach an efficacy of 70% with risks of hepatotoxicity and bone marrow suppression (12).

As biologics come to the stage in the therapy of autoimmune disease, studies showing favorable results of adalimumab in uveitis treatment are accumulating. Randomized controlled trials (RCTs), such as the VISUAL trial series, have shown that adalimumab could significantly decrease the risk of uveitic flare in adult patients with active and inactive noninfectious intermediate, posterior, or panuveitic uveitis (1, 2). The SYCAMORE and ADJUVITE studies focused on the pediatric group and have shown that adalimumab could effectively control inflammation and was associated with a lower rate of treatment failure in JIA-associated anterior uveitis (3, 13). However, the timing and the specific indication for starting adalimumab remain to be further addressed. A recently published study showed that 80% of pediatric idiopathic uveitis manifests a certain degree of retinal vasculitis, which is associated with a lower probability of inflammation control, more intensive treatment, and worse visual prognosis (14). To the best of our knowledge, there is no specific study examining the efficacy of adalimumab in the treatment of pediatric uveitis with vascular leakage.

In this study, pediatric patients suffered from chronic inflammation in which topical GC could not be tapered, even though some patients were treated with concomitant systemic IMT. These patients shared a characteristic of peripheral retinal vascular leakage in UWFFA, which was reported to be significantly correlated with anterior chamber cells, vitreous cells (15), cystoid macular edema (16, 17), and treatment augmentation (18, 19). Our experiences were in line with these studies that vascular leakage on UWFFA might be a disease severity monitoring sign independent of conventional measurements (i.e., anterior chamber cells, vitreous cells, etc.), indicative of chronicity, and refractoriness in pediatric anterior uveitis. The efficacy of adalimumab for children with this particular finding was evaluated in this study, and the alleviation of vascular leakage was set as the primary outcome.

As expected, vascular leakage, represented by the ASUWOG score, is reduced dramatically upon the addition of adalimumab, which is intuitively demonstrated in Figure 2. Apparently, the reduction in vascular leakage on UWFFA was in parallel with other traditional measurements of disease activity. However, when we tried to perform a correlation test to demonstrate this association, we did not obtain a definite result (data not shown), as expected. The reason might come from three aspects. One was that the change in the traditionally measured inflammatory parameters was similar in each individual, but the change in the extent of vascular leakage was of a wider range. Thus, a statistically significant correlation was hard to determine. Another aspect might be caused by the time interval between the traditional inflammatory parameter data collection and the performance of UWFFA. In the real clinical setting, potential candidates for adalimumab application would undergo UWFFA screening first, and further blood test results or X-ray were needed before the application of adalimumab, when the other inflammatory parameters were collected. Third, for ethical concerns, if retinal vascular leakage healed without relapse of anterior chamber inflammation, UWFFA at 6 months was not performed. Thus, data were limited to fully demonstrate the correlations between peripheral retinal vascular leakage and the other inflammatory parameters. Due to these concerns, we still held a positive view that peripheral vascular leakage on UWFFA might serve as an indicator of disease activity and be useful for disease monitoring in pediatric anterior uveitis.

In this study, as inflammation was controlled, prolongation of adalimumab administration was attempted. Most patients could remain inflammatory quiescent after this dose reduction, indicating that there might be a possibility that chronic pediatric anterior uveitis could be controlled at least temporally by adalimumab treatment. However, due to the retrospective nature of our study, the adalimumab administration interval was adjusted based on the clinicians' experience and was not standardized. Currently, there is no international consensus on how long adalimumab should be used, specifically for pediatric chronic anterior uveitis with peripheral vascular leakage. We represented our treatment condition in a real-world setting only to provide more evidence that some children could tolerate adalimumab prolongation after several months of treatment. However, when adalimumab could be tapered remained to be further investigated.

Despite this study, all children with peripheral vascular leakage responded well to adalimumab.

There were several studies demonstrating that some refractory uveitis was also resistant to adalimumab (20), which might be caused by anti-adalimumab antibody formation (21, 22) and the nature of the disease. In this study, pediatric patients with chronic anterior uveitis accompanied by peripheral vascular leakage were selected. For this specific group of patients, we provided good evidence that adalimumab could effectively alleviate peripheral vascular leakage while simultaneously controlling intraocular inflammation. We must admit that the sample size was relatively small to determine if there was any possibility of refractoriness for this specific group of patients, and the enrolled population was homogeneous (mainly Han Chinese children) to provide generalized conclusions. We would like to accumulate more cases in the future to evaluate the refractory rate and rescue plans for this group of patients.

Our study also revealed a favorable safety profile of adalimumab in Chinese children with anterior uveitis. Only mild adverse effects, such as local redness around the injection site and mild upper respiratory infection, were reported, which was comparable to the safety profile derived from 23,458 adult patients in 71 global RCTs and 577 pediatric patients in seven global RCTs (23, 24).

Several limitations of this study should also be addressed. First, our study was a retrospective self-controlled study and only included patients with peripheral retinal vascular leakage on UWFFA; therefore, we were unable to compare them to those without. Performing UWFFA in children with nonchronic and nonrefractory uveitis is not ethical; therefore, we have extremely limited data on pediatric uveitis without vascular leakage to perform a case–control study. Due to the retrospective nature of the study, topical eye drops, and systemic IMT adjustment, the adalimumab prolongation method was based on the clinicians' clinical experience and therefore not standardized. A future prospective RCT is needed to confirm the superiority of adalimumab over other traditional IMTs. Second, all the children in this study were from the Chinese Han population, and the relative homogeneity of the cohort does not allow us to generalize our findings to other ethnic groups. Third, the follow-up period was relatively short to monitor the long-term efficacy of adalimumab and its safety profile. Patients in this study will be followed up longer for more convincing long-termevidence.

In conclusion, adalimumab used in pediatric chronic uveitis could effectively alleviate peripheral retinal vascular leakage. The application of adalimumab could also help improve BCVA, control intraocular inflammation, and taper the dose of topical GC eye drops and IMT.

## Data Availability Statement

The raw data supporting the conclusions of this article will be made available by the authors, without undue reservation.

## Ethics Statement

The studies involving human participants were reviewed and approved by the Institutional Review Board of Peking Union Medical College Hospital. Written informed consent to participate in this study was provided by the participants' legal guardian/next of kin.

## Author Contributions

MZ, CZ, and HS designed the study. MZ, CZ, and FG recruited patients. DL performed all the UWFFA examinations. HS collected data, performed the analysis, and wrote the manuscript. HS and JX were responsible for the ASUWOG scoring. CZ, MZ, and JX critically reviewed, edited, and finalized the manuscript for submission. All authors contributed to the article and approved the submitted version.

## Funding

This work was supported by the Nonprofit Central Research Institute Fund of the Chinese Academy of Medical Sciences (2018PT32029).

## Conflict of Interest

The authors declare that the research was conducted in the absence of any commercial or financial relationships that could be construed as a potential conflict of interest.

## Publisher's Note

All claims expressed in this article are solely those of the authors and do not necessarily represent those of their affiliated organizations, or those of the publisher, the editors and the reviewers. Any product that may be evaluated in this article, or claim that may be made by its manufacturer, is not guaranteed or endorsed by the publisher.
